# Supplementary material for: Drug repurposing for aging research using model organisms
Source: Aging Cell. 2017 Jun 16;16(5):1006–15. doi: 10.1111/acel.12626 (PMC5595691; doi:10.1111/acel.12626)
Supplement: Supplementary file 7 — Data S1 Zip‐Archive of all report cards. [file ACEL-16-1006-s007.zip › RC_MLA.pdf]

MLA

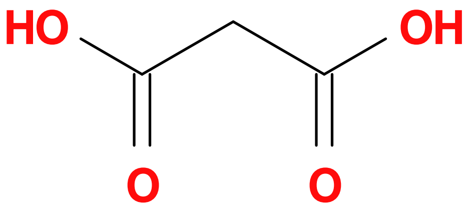

Database identifiers

ChEMBLCompound	CHEMBL7942
DrugBank	DB02175
CHEBI	30794
eMolecules	494228

Ranking

	Rank	Score
Drosophila	508/697	0.259
C. elegans	292/591	0.196

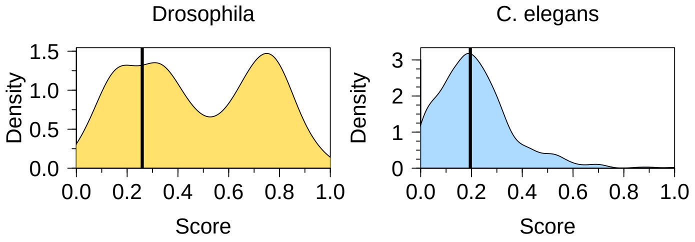

	Ageing implication	Domain conservation	Binding site conservation	Binding affinity	Bioavailability	Lipinski	Promiscuity	Purchasability	Drug approval	**Total**
Drosophila	0.36	0.973	1.0	0.272	(0.9)	0.0	-0.001	0.1	0.075	0.259
C. elegans	0.36	0.97	1.0	0.272	0.23	0.0	-0.001	0.1	0.075	0.196

Names

- **malonic acid**
- H2malo
- Hooc-ch2-cooh
- Propanedioic acid

Roles

human metabolite

Status

Approved drug (according to ChEMBL)	No
Classification (according to DrugBank)	experimental
Number of Rule of 5 violations	0
Binding affinity to original target in log units (RF-Score prediction)	4.02
Burns *C. elegans* bioavailability prediction	-3.31

Compound Target Characteristics

Glycogen synthase kinase-3 beta

Best gene implication in ageing for this target family came from gene D3Z7E5 via mapping the annotation from Ensembl ENSMUSG00000057177 via mapping the annotation from EntrezGene 606496 via mapping the annotation from GenAgeModels 1807 annotated in GenAge release 17.

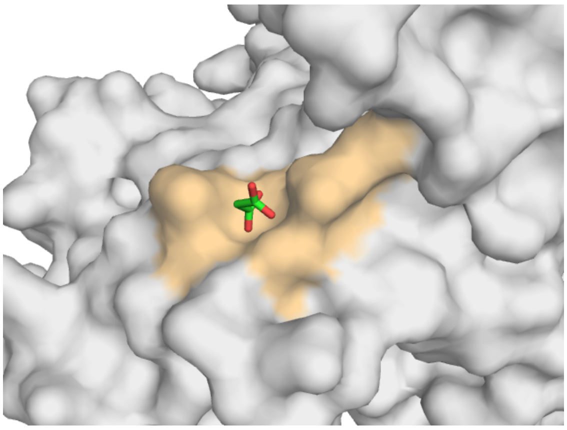

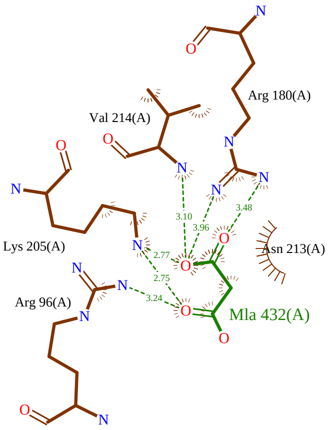

protein	amino acids contacts (binding site)
PDB:3say:chainA:P49841	R R K N V
tr:Q68D16:Q68D16_HUMAN	R R K N V
tr:A8MT37:A8MT37_HUMAN	R R K N V
tr:Q6FI27:Q6FI27_HUMAN	R R K N V
sp:P49841:GSK3B_HUMAN	R R K N V
sp:P49840:GSK3A_HUMAN	R R K N V
sp:P18266:GSK3B_RAT	R R K N V
tr:Q5KU03:Q5KU03_MOUSE	R R K N V
sp:Q9WV60:GSK3B_MOUSE	R R K N V
tr:E9QAQ5:E9QAQ5_MOUSE	R R K N V
tr:D3Z7E5:D3Z7E5_MOUSE	R R K N V
sp:Q2NL51:GSK3A_MOUSE	R R K N V
tr:B6IDN4:B6IDN4_DROME	R R K N V
sp:P83101:GSK3H_DROME	R R K N V
sp:P18431:SGG_DROME	R R K N V
tr:A4V3W2:A4V3W2_DROME	R R K N V
tr:A4V3W1:A4V3W1_DROME	R R K N V
tr:Q0KHW6:Q0KHW6_DROME	R R K N V
tr:A8JUV9:A8JUV9_DROME	R R K N V
tr:E6PBX9:E6PBX9_DROME	R R K N V
tr:H5V852:H5V852_DROME	R R K N V
sp:Q9U2Q9:GSK3_CAEEL	R R K N V
sp:P38615:RIM11_YEAST	R R K N V
sp:P50873:MRK1_YEAST	R R K N V

protein	whole protein ident	simil	domain-based ident	simil	contact-based ident	simil
PDB:3say:chainA:P49841	1.0	1.0	1.0	1.0	1.0	1.0
tr:Q68D16:Q68D16_HUMAN	0.74	0.86	0.9	0.97	1.0	1.0
tr:A8MT37:A8MT37_HUMAN	0.72	0.84	0.9	0.97	1.0	1.0
tr:Q6FI27:Q6FI27_HUMAN	1.0	1.0	1.0	1.0	1.0	1.0
sp:P49841:GSK3B_HUMAN	1.0	1.0	1.0	1.0	1.0	1.0
sp:P49840:GSK3A_HUMAN	0.65	0.79	0.9	0.97	1.0	1.0
sp:P18266:GSK3B_RAT	0.99	1.0	1.0	1.0	1.0	1.0
tr:Q5KU03:Q5KU03_MOUSE	0.99	1.0	1.0	1.0	1.0	1.0
sp:Q9WV60:GSK3B_MOUSE	0.99	1.0	1.0	1.0	1.0	1.0
tr:E9QAQ5:E9QAQ5_MOUSE	0.96	0.97	0.96	0.96	1.0	1.0
tr:D3Z7E5:D3Z7E5_MOUSE	0.64	0.77	0.89	0.96	1.0	1.0
sp:Q2NL51:GSK3A_MOUSE	0.64	0.78	0.89	0.96	1.0	1.0
tr:B6IDN4:B6IDN4_DROME	0.58	0.71	0.85	0.96	1.0	1.0
sp:P83101:GSK3H_DROME	0.46	0.65	0.72	0.93	1.0	1.0
sp:P18431:SGG_DROME	0.58	0.73	0.85	0.96	1.0	1.0
tr:A4V3W2:A4V3W2_DROME	0.58	0.73	0.85	0.96	1.0	1.0
tr:A4V3W1:A4V3W1_DROME	0.52	0.65	0.85	0.96	1.0	1.0
tr:Q0KHW6:Q0KHW6_DROME	0.49	0.63	0.85	0.96	1.0	1.0
tr:A8JUV9:A8JUV9_DROME	0.34	0.43	0.77	0.84	1.0	1.0
tr:E6PBX9:E6PBX9_DROME	0.18	0.25	0.52	0.65	1.0	1.0
tr:H5V852:H5V852_DROME	0.28	0.35	0.85	0.96	1.0	1.0
sp:Q9U2Q9:GSK3_CAEEL	0.62	0.78	0.81	0.95	1.0	1.0
sp:P38615:RIM11_YEAST	0.43	0.7	0.57	0.86	1.0	1.0
sp:P50873:MRK1_YEAST	0.31	0.57	0.53	0.84	1.0	1.0

gskt (FBgn0046332) associated phenotypes

male sterile germline-dependent

(Information from FlyBase)

gskt (UniProt:P83101) annotation

Ptm: Phosphorylation on Tyr-193 is necessary for the activity.

(Information from UniProt)

sgg (FBgn0003371) associated phenotypes

cell non-autonomous, cell polarity defective, chemical sensitive, circadian rhythm defective, developmental rate defective, germline clone, heat sensitive, lethal - all die before end of P-stage, lethal - all die before end of pupal stage, maternal effect, meiotic cell cycle defective, mitotic cell cycle defective, neuroanatomy defective, neurophysiology defective, non-rescuable maternal effect, planar polarity defective, rescuable maternal effect, short lived, smell perception defective, somatic clone, some die during pharate adult stage, some die during pupal stage, some die during third instar larval stage

(Information from FlyBase)

sgg (UniProt:P18431) annotation

Function: Required for several developmental events such as syncytial blastoderm formation and embryonic segmentation. Is involved in transcriptional regulation. Required for arm phosphorylation. Wg signaling operates by inactivating the sgg repression of en autoactivation. Negatively controls the neuromuscular junction (NMJ) growth in presynaptic motoneurons. Plays a role in the regulation of microtubule dynamics and actin cytoskeleton during embryogenesis. Required for phosphorylation of sra in activated eggs. Essential for completion of meiosis, possibly by triggering calcineurin activation via sra phosphorylation. Phosphorylates microtubule-associated protein futsch in axons. (PubMed:15269269, PubMed:16570248, PubMed:16949836, PubMed:2113617, PubMed:2118107, PubMed:22421435, PubMed:7529201, PubMed:8467811).

Subunit: Interacts with cos. (PubMed:1335365,PubMed:15691767).

Subcellular location: Cytoplasm. Nucleus. Cytoplasm, cytoskeleton, microtubule organizing center, centrosome. Cytoplasm, cell cortex. Cell junction, synapse. Cell projection, axon. Note=In syncytial embryos, detected at the centrosomes throughout the cell cycle, and in the mitotic spindle

and pseudocleavage furrows invaginating from the cell cortex during mitosis. Concentrated at the growing end of membranes during the cellularization process. After cellularization, localized to the centrosomes during mitosis and to the nucleus at the end of telophase. Enriched in the presynaptic side of the neuromuscular junction, with some signal detected also in axonal branches.
Tissue specificity: Expressed in ovaries and activated eggs (at protein level). Expression is over all the embryo at all stages, no local accumulation is observed. (PubMed:2118107, PubMed:22421435, PubMed:8467811).
Developmental stage: Isoform SGG46 is expressed at low levels in 12-24 hours embryos. Isoform Zygotic and isoform SGG39 are expressed in 12-24 hours embryos and present throughout the larval, pupal and adult stages (at protein level). Isoform Zygotic is expressed maternally and zygotically but reduced throughout later embryonic development. Expression persists throughout larval stages. (PubMed:2118107, PubMed:8467811).
Disruption phenotype: Mutants display an overdeveloped neuromuscular junction (NMJ), with the number of boutons greatly increased. (PubMed:15269269).

(Information from UniProt)

gsk-3 (WBGene00001746) associated phenotypes

axon morphology variant, larval lethal, lethal, locomotion variant, sterile

(Information from WormBase)

gsk-3 (UniProt:Q9U2Q9) annotation

Function: Phosphorylates oma-1, a regulator of the oocyte-to- embryo transition, enabling its degradation. Phosphorylates skn-1, preventing it from accumulating in nuclei and thus inhibiting phase II gene expression in the oxidative stress defense. Involved in mesendoderm specification and mitotic spindle orientation in EMS blastomeres. Thought to be a branch point in these processes as proteins downstream are not required. Negatively regulates Wnt signaling in vulval precursor cells and acts as a Wnt-independent repressor of med-1 and med-2 in the C lineage inhibiting mesoderm development. Required for normal lifespan and LiCl-induced lifespan extension. (PubMed:10444600, PubMed:11463373, PubMed:15572126, PubMed:16251270, PubMed:16289132, PubMed:16343905, PubMed:16930586, PubMed:17959600).
Subunit: Monomer (By similarity). Interacts with axl-1. (UniProtKB:Q9WV60, PubMed:17601533).
Disruption phenotype: Worms exhibit defects in endoderm specification and mitotic spindle alignment. Mutants show reduced degradation of oma-1 and have shortened lifespan. (PubMed:10444600).

(Information from UniProt)

Protein deglycase DJ-1

Best gene implication in ageing for this target family came from gene Q6NMT9 via mapping the annotation from Ensembl FBgn0033885 via mapping the annotation from EntrezGene 36543 via mapping the annotation from GenAgeModels 0178 annotated in GenAge release 17.

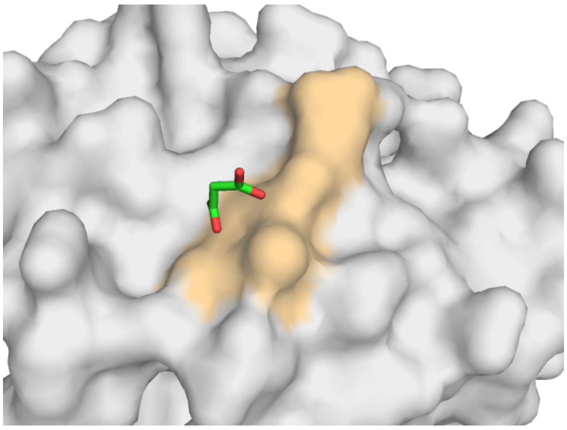

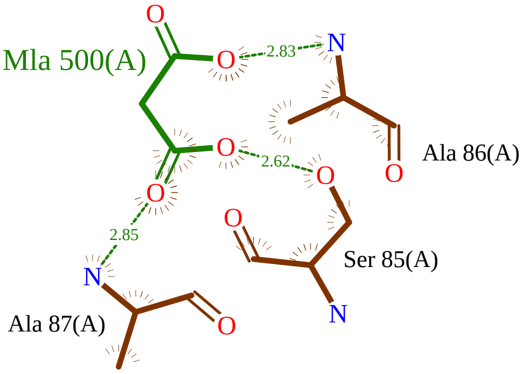

protein	amino acids contacts (binding site)			
PDB:3cza:chainA:Q99497	E	S	A	A
tr:K7ELW0:K7ELW0_HUMAN	E	S	A	A
tr:K7EN27:K7EN27_HUMAN	E	S	A	A
sp:Q99497:PARK7_HUMAN	E	S	A	A
sp:O88767:PARK7_RAT	E	S	A	L
tr:M0R3K9:M0R3K9_RAT	E	S	A	M
tr:A2A816:A2A816_MOUSE	E	V	K	A
tr:A2A815:A2A815_MOUSE	E	S	P	M
tr:A2A813:A2A813_MOUSE	E	S	P	M
sp:Q99LX0:PARK7_MOUSE	E	S	P	M
tr:Q6NMT9:Q6NMT9_DROME	N	S	S	A
tr:Q9VA37:Q9VA37_DROME	E	S	S	L
tr:A1Z9J4:A1Z9J4_DROME	N	S	S	A
tr:P90994:P90994_CAEEL	E	S	L	L
tr:O16228:O16228_CAEEL	E	C	P	V

	whole protein		domain-based		contact-based	
protein	ident	simil	ident	simil	ident	simil
PDB:3cza:chainA:Q99497	0.99	1.0	0.99	1.0	1.0	1.0
tr:K7ELW0:K7ELW0_HUMAN	0.89	0.89	0.89	0.89	1.0	1.0
tr:K7EN27:K7EN27_HUMAN	0.75	0.75	0.76	0.76	1.0	1.0
sp:Q99497:PARK7_HUMAN	1.0	1.0	1.0	1.0	1.0	1.0
sp:O88767:PARK7_RAT	0.92	0.98	0.91	0.98	0.75	0.78
tr:M0R3K9:M0R3K9_RAT	0.67	0.81	0.74	0.9	0.75	0.8
tr:A2A816:A2A816_MOUSE	0.48	0.57	0.48	0.58	0.5	0.47
tr:A2A815:A2A815_MOUSE	0.67	0.7	0.67	0.71	0.5	0.74
tr:A2A813:A2A813_MOUSE	0.83	0.9	0.84	0.91	0.5	0.74
sp:Q99LX0:PARK7_MOUSE	0.92	0.98	0.91	0.98	0.5	0.74
tr:Q6NMT9:Q6NMT9_DROME	0.35	0.5	0.42	0.6	0.5	0.67
tr:Q9VA37:Q9VA37_DROME	0.47	0.74	0.52	0.82	0.5	0.55
tr:A1Z9J4:A1Z9J4_DROME	0.46	0.69	0.54	0.81	0.5	0.67
tr:P90994:P90994_CAEEL	0.52	0.82	0.52	0.82	0.5	0.55
tr:O16228:O16228_CAEEL	0.43	0.78	0.44	0.79	0.25	0.59

dj-1beta (FBgn0039802) associated phenotypes

chemical resistant, chemical sensitive, locomotor behavior defective, long lived, oxidative stress response defective, radiation sensitive, short lived, stress response defective

(Information from FlyBase)

DJ-1alpha (FBgn0033885) associated phenotypes

chemical resistant, chemical sensitive, increased cell death, locomotor behavior defective, short lived, stress response defective

(Information from FlyBase)

djr-1.1 (WBGene00015184) associated phenotypes
solitary feeding increased

(Information from WormBase)

djr-1.1 (UniProt:P90994) annotation
Function: Catalyzes the conversion of methylglyoxal (MG) or glyoxal (GO) to D-lactate or glycolic acid respectively in a single glutathione (GSH)-independent step. May play a role in detoxifying endogenously produced glyoxals. Involved in protection against glyoxal-induced cell death. (PubMed:22523093).
Biophysicochemical properties: Kinetic parameters: KM=0.3 mM for methylglyoxal (PubMed:22523093); KM=3.61 mM for glyoxal (PubMed:22523093); Note=kcat is 13.8 min(-1) with methylglyoxal as substrate and 356.4 min(-1) with glyoxal as substrate. (PubMed:22523093);
Subcellular location: Cytoplasm (PubMed:22523093). Nucleus (PubMed:22523093). Note=Ubiquitously localized throughout the whole cells with higher expression in the nucleus. (PubMed:22523093).
Tissue specificity: Expressed exclusively in the intestine. (PubMed:22523093).

(Information from UniProt)

djr-1.2 (UniProt:O16228) annotation
Function: Catalyzes the conversion of methylglyoxal (MG) or glyoxal (GO) to D-lactate or glycolic acid respectively in a single glutathione (GSH)-independent step. May play a role in detoxifying endogenously produced glyoxals. Involved in protection against glyoxal-induced cell death. Protects dopaminergic neurons from glyoxal-dependent neuronal degeneration. (PubMed:22523093, PubMed:23624124).
Biophysicochemical properties: Kinetic parameters: KM=0.39 mM for methylglyoxal (PubMed:22523093); KM=0.78 mM for glyoxal (PubMed:22523093); Note=kcat is 60.0 min(-1) with methylglyoxal as substrate and 146.4 min(-1) with glyoxal as substrate. (PubMed:22523093);
Subcellular location: Cytoplasm (PubMed:22523093).
Tissue specificity: Expressed in various tissues, including pharyngeal muscles, pharynx-intestinal valve, ventral nerve cord, spermatheca, rectal gland, inner labial (IL) cells of head neurons, phasmid (PHA/PHB) neurons in tail and supporting sheath/socket cells, as well as in head mesodermal cells (HMC), excretory canals and coelomocytes. (PubMed:22523093).
Induction: Induced by DAF-16 during starvation as well as in the dauer stage. (PubMed:23624124).

(Information from UniProt)
